# Computational Modelling of the Interactions Between Polyoxometalates and Biological Systems

**DOI:** 10.3389/fchem.2022.876630

**Published:** 2022-04-14

**Authors:** Adrià Gil, Jorge J. Carbó

**Affiliations:** ^1^ ARAID Foundation, Zaragoza, Spain; ^2^ Departamento de Química Inorgánica, Instituto de Síntesis Química y Catálisis Homogénea (ISQCH) CSIC, Universidad de Zaragoza, Zaragoza, Spain; ^3^ Faculdade de Ciências, BioISI—Biosystems and Integrative Sciences Institute, Universidade de Lisboa, Lisboa, Portugal; ^4^ Department de Química Física i Inorgànica, Universitat Rovira i Virgili, Tarragona, Spain

**Keywords:** polyoxometalate, computational chemistry, protein, artificial enzymes, molecular dynamics, DFT, peptide hydrolysis, phosphoester hydrolysis

## Abstract

Polyoxometalates (POMs) structures have raised considerable interest for the last years in their application to biological processes and medicine. Within this area, our mini-review shows that computational modelling is an emerging tool, which can play an important role in understanding the interaction of POMs with biological systems and the mechanisms responsible of their activity, otherwise difficult to achieve experimentally. During recent years, computational studies have mainly focused on the analysis of POM binding to proteins and other systems such as lipid bilayers and nucleic acids, and on the characterization of reaction mechanisms of POMs acting as artificial metalloproteases and phosphoesterases. From early docking studies locating binding sites, molecular dynamics (MD) simulations have allowed to characterize the nature of POM···protein interactions, and to evaluate the effect of the charge, size, and shape of the POM on protein affinity, including also, the atomistic description of chaotropic character of POM anions. Although these studies rely on the interaction with proteins and nucleic acid models, the results could be extrapolated to other biomolecules such as carbohydrates, triglycerides, steroids, terpenes, etc. Combining MD simulations with quantum mechanics/molecular mechanics (QM/MM) methods and DFT calculations on cluster models, computational studies are starting to shed light on the factors governing the activity and selectivity for the hydrolysis of peptide and phosphoester bonds catalysed by POMs.

## Introduction

Polyoxometalates (POMs) are a unique class of well−defined polynuclear metal oxide clusters that are usually built up from early transition metal ions such as W, Mo or V in their highest oxidation state with an overwhelming diversity in size, composition and structures. They have been widely applied in catalysis due to their tunable Brønsted acidity combined with redox properties, as well as, their ability to accommodate other transition metals (TMs) in their structure. (Wang and Yang, 2015). In addition, polyoxometalates are water-soluble, large anions that give rise to unusual solution behavior that have found applications in biotechnology and supramolecular chemistry. (Arefian et al., 2017; van Rompuy and Parac-Vogt, 2019; Aureliano et al., 2021). Recently, this behavior has been attributed to the (super)chaotropic character of the POMs anions, whose low charge density make them to be weakly hydrated, and consequently, exhibit propensity to assemble with organic moieties and biomolecules (see below for a deeper description). (Assaf and Nau, 2018). Among the applications in biotechnology and medicine, POMs have shown *in vitro* and *in vivo* antiviral, antibacterial or antitumor properties; utility in protein crystallography; or activity as artificial metalloproteases and phosphoesterases. (Aureliano et al., 2021), (Bijelic et al., 2018; Bijelic et al., 2019; Pessoa et al., 2021; Aureliano et al., 2022) (Bijelic et al., 2018; Bijelic et al., 2019; Pessoa et al., 2021; Aureliano et al., 2022).

The bioactivity of POMs depends largely on their ability to establish specific interactions with biomolecules, and therefore, the precise understanding of these interactions is crucial for further developments. Moreover, to exploit the use of metal-substituted POMs as a novel class of artificial enzymes, it is necessary to progress in characterizing and rationalizing their mechanism of action. Previous reviews in computational POM chemistry have mainly collected studies on structure, electronic properties, spectroscopy, and reactivity based on Density Functional Theory methods (DFT) in conjuction with continuous solvent models. (López et al., 2011; López et al., 2012). More recently, the development of tailor-made, classical potentials for POMs has allowed to perform dynamic simulations, such as Molecular Dynamics (MD), on complex (bio)molecular systems including dynamic properties and explicit solvent effects. Here, we focus on how recent computational studies have contributed to increase understanding of the physicochemical foundations underlying the basic principles of the observed biological activity of POMs.

## Interaction Between Polyoxometalates and Biomolecules

**FIGURE 1:**
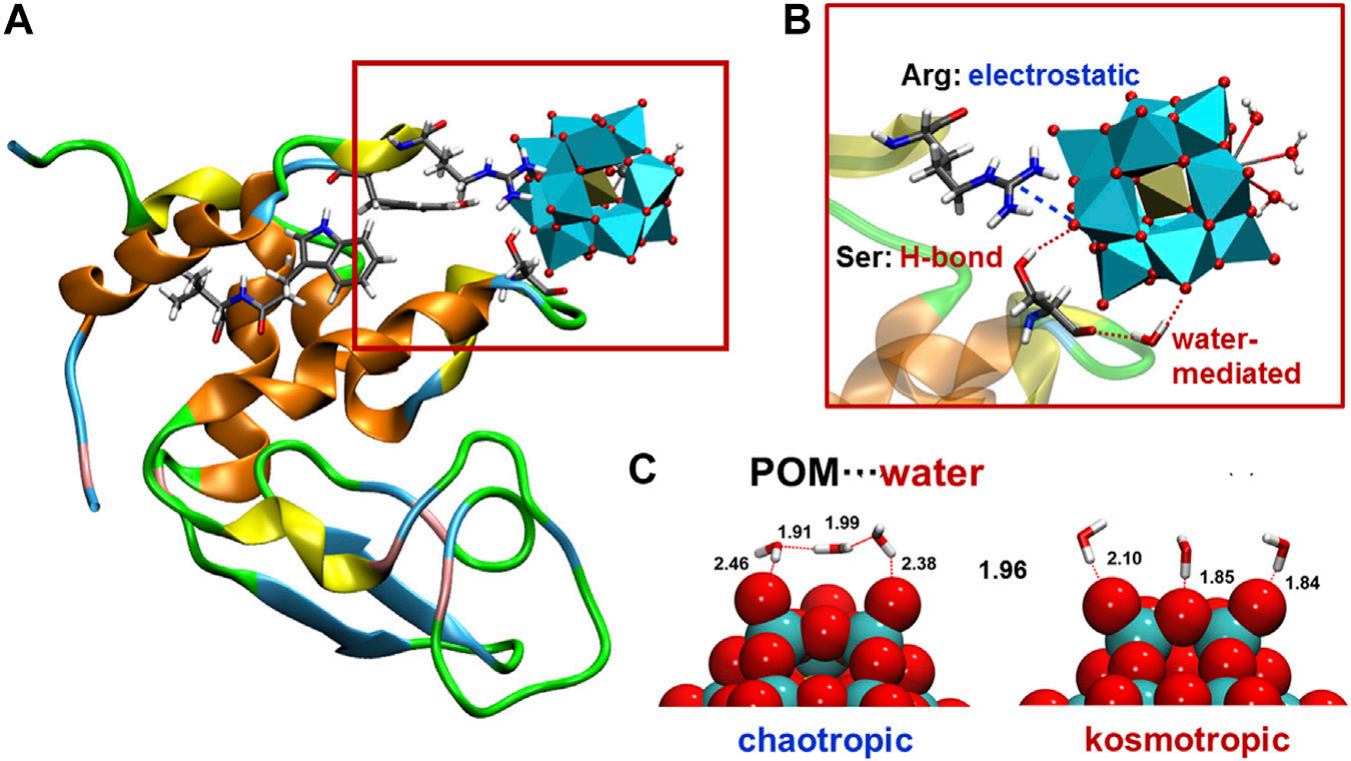
**(A)** Illustrative snapshot of the interaction between HEWL protein and Ce-substituted Keggin POM [ref (Solé-Daura et al., 2016)]; **(B)** closer look highlighting interaction with individual amino acids of different nature type (electrostatic, hydrogen-bonding, and water-mediated); **(C)** representative snapshot of the water distribution around two POMs of different charge density [moderately and highly charged for chaotripic and komostropic POM···water interaction, respectively, ref (Solé-Daura et al., 2020a)].

The interaction of POMs with proteins have attracted attention of many scientists because it plays a crucial role in mechanistic pathways governing the biological activity exhibited by POMs. (Aureliano et al., 2021). Although covalent interaction between biomolecules and POMs have been reported, (Molitor et al., 2016), most of these interactions are of non-bonding nature. Experimentally, there are limitations to identify the binding modes and characterise the nature of these interaction, as well as to assess the competition for binding sites between POMs and other species in the media. Computationally, early docking studies explored the binding locations showing that POMs interact mainly at positively charged patches of the protein, where cationic- and polar-type amino acids predominate. (Narasimhan et al., 2011; Prudent et al., 2010; Prudent et al., 2008; Hu et al., 2007; Tiago et al., 2007; Pezza et al., 2002; Judd et al., 2001; Sarafianos et al., 1996). Owing to the intrinsic limitations of docking methods, atomistic molecular dynamics (MD) simulations have been more recently performed to reveal the driving forces that are responsible for the specific interactions (Figure 1A)[ (Solé-Daura et al., 2016; Paul et al., 2018; Solé-Daura et al., 2020a; Sciortino et al., 2021; Chaudhary et al., 2021)] Pioneer MD simulations analysed the interaction between model protein hen egg-white lysoszyme (HEWL) and three different POMs, the Ce-substituted Keggin-type anion [PW_11_O_39_Ce(OH_2_)_4_]^3−^ the corresponding 1:2 dimer [Ce(PW_11_O_39_)_2_]^10−^ and the Zr-substituted Lindqvist-type anion [W_5_O_18_Zr(OH_2_) (OH)]^3−^, which differ in the overall charge, the size, the shape and the type of substituted metal. (Solé-Daura et al., 2016). All POM structures interacted preferentially with positively charged Lys- and Arg-rich patches on the protein surface. The nature of these interactions comprises mainly electrostatic attraction, hydrogen bonding and water-mediated interactions, not only with positively-charged amino acids such as lysine and arginine, but also with uncharged polar amino acids such as tyrosine, serine and asparagine (Figure 1B). The basic oxygen atoms of POM framework interact with the side chains of the amino acids and, in lesser extent, with the N-H amide group of the main protein chain. Moreover, depending on the size and shape of the POM, several amino acids can interact simultaneously with the oxide framework, favoring the formation of POM-protein complexes. These results were subsequently backed by X-ray structural characterization of non-covalent complexes between HEWL and several transition metal-substituted tungstates. This also suggests that the interaction is largely independent on the nature of substituted metal within the same polyoxometalate structure type because the interaction occurs through the POM oxide framework. (Sap et al., 2015; Vandebroek et al., 2019). Interactions of the same nature were computationally characterised for the binding of Zr-substituted Keggin tungstates to human serum albumin (HSA) protein. (Paul et al., 2018).

The attempts to set structure-activity relationships for the affinity of POMs towards proteins, and then relate them to their biological activity, have some limitations from the experimental point of view. For example, they include the lack of stability under experimental conditions, or the limited number of structures available making them to differ in more than one feature at a time. On the other hand, computational modelling allows performing systematic variations on single parameters of well-defined POM structures. Thus, a fundamental study based on MD simulations and descriptor-based modeling has been recently reported, in which the charge or the size and shape of a series of tungstate POMs were modified systematically and the affinity towards HEWL protein evaluated. (Solé-Daura et al., 2020a). Using two molecular descriptors that account for the charge density (charge per metal atom ratio; *q/M*) and the size and shape (shape-weighted volume; *V*_*S*_), it was possible to build quantitative multidimensional regression models for protein affinity with predictive ability. Interestingly, the model reveals the non-linear relationship between protein affinity and both the charge density and the size of the POM, as a result of a delicate balance between POM···protein and POM···solvent interactions. Atomistic simulations revealed the variation of hydrogen bonding patterns for POM···water interactions depending on POM charge density. Moderately charged POMs anions have chaotropic character (water-structure-breaking), while highly charged POMs anions have kosmotropic properties (water-structure-forming) which results in proportionally larger desolvation energies, and consequently, less affinity towards proteins (Figure 1C). In fact, Nau and co-workers have defined POMs as superchaotropic anions that extent beyond the classical Hofmeister scale; that is, low charge density anions that are weakly hydrated and consequently have propensity to assemble with organic moieties and biomolecules. (Assaf and Nau, 2018). These simulations also showed that POM···protein interactions are size-specific, and that Keggin-type anions have the optimal size and shape to fit the cationic sites of HEWL. (Solé-Daura et al., 2020a).

More recently, computational studies based on MD simulations have also analysed the interaction of polyoxoniobates and polyoxovanadates with different proteins. (Sciortino et al., 2021), (Chaudhary et al., 2021) The nature of the characterised interactions are similar to those of tungstates and transition metal-substituted tungstates with the POM···protein binding dominated by electrostatic and hydrogen bonding forces. Experimentally, the analysis of X-ray polyoxovanadate-protein structures show that the binding sites include a variety of positively charged amino acids such as Arg, His and Lys. (Aureliano et al., 2022). Computationally, a comparison of decavanadate anion (V_10_O_28_^6−^) with the isostructural and equally charged decaniobate (Nb_10_O_28_^6−^) was carried out in their interplay with the globular actin protein. (Sciortino et al., 2021). Interestingly, they prefer different binding sites of the protein (the catalytic nucleotide site α for V_10_O_28_^6−^ and the β site for Nb_10_O_28_^6−^), both inducing conformational arrangements in the protein, suggesting that biological activity could be synergistic. MD calculations confirmed that polyoxoniobates [Nb_10_O_28_]^6−^ and [TiNb_9_O_28_]^7−^ interact with positively charged sites of the surface of native S100A9 protein, which is a pro-inflammatory and amyloidogenic protein involved in neurodegenerative diseases, interacting simultaneously with several amino acids at a highly dynamic part of the protein. (Chaudhary et al., 2021). Experimentally, these two POMs acted as inhibitors of S100A9 amyloid assembly. (Chaudhary et al., 2021). Additionally, the same authors assessed how the ionic strength of the media influences the complex formation. Increasing the NaCl salt concentration from 20 to 150 mM in the simulated system reduced the formation of POM-protein complexes.

The interaction of POMs with protein surfaces can induce changes on its structure. For example the dimeric 1:2 POM K_16_ [Hf(α_2_-P_2_W_17_O_61_)_2_] co-crystalise with HEWL in its monomeric form, which was never observed in water solution. (Vandebroek et al., 2018). This indicates that the dimeric POM dissociates upon binding because it moves from the highly polar bulk water to the protein surface which has a lower polarity (lower dielectric constant). DFT calculations with continuum solvent model evaluated the free energy cost of dissociation process at different dielectric constants that is unfavorable at the dielectric constant of bulk water (ε_r_ = 80) and it becomes favorable at lower dielectric constants (ε_r_ = 20–50). (Vandebroek et al., 2018). Thus, theoretical calculations support the protein-assisted dissociation of group IV transition metal-substituted dimeric structures. It is to note that computational methods have studied other factors influencing the dissociation of these dimers such as the pH or concentration (Jiménez-Lozano et al., 2014; Jiménez-Lozano et al., 2017). There have been also several molecular modeling studies on POM-oligopeptide hybrids, in which the polypeptide is covalently linked to the polyoxometalate. (Vilona et al. 2018; Nikoloudakis et al 2018). Besides their own interest as potential drugs or as building blocks for self-assembled materials, these compounds can serve as more tractable structures, allowing to combine classical MD with quantum mechanics DFT calculations, in order to understand the specific interactions of peptides with the metal-oxide surface of the POM. For example, in tin-substituted Dawson polyoxotungstates, the polyglycine side chains folds towards the metal-oxide surface forming zipper hydrogen bond networks. (Vilona et al., 2018). Interestingly, the intramolecular hydrogen bonds are formed preferentially with the terminal oxygens (W=O), even that they are less basic than the W-O-W bridging μ_2_ oxygens. Even classical molecular dynamics simulations present limitations since they cannot handle very big and complex systems such as lipid bilayers, and therefore this type of systems have been scarcely studied. Nevertheless, by sacrificing molecular details, coarse-grained MD simulations analysed the embedding of a giant POM (Mo_132_ type Keplerate capsule) in lipid bilayer membranes. (Carr et al., 2008). The simulated system remained stable, and water was observed to flow into and out of the capsule as well as Na^+^ cations, suggesting that Mo_132_ can form a functional synthetic ion channel.

## Polyoxometalates as Artificial Enzymes

The ability of POMs to form specific interactions with biomolecules combined with their capacity to catalyse reactions have prompted their use as artificial metalloenzymes. Computationally, two main processes have been analysed: 1) the peptide bond hydrolysis in di-, oligopeptides and proteins with potential applicability to proteomics, and 2) the phosphoester bond hydrolysis.

### Mechanism of Peptide Bond Hydrolysis

**FIGURE 2:**
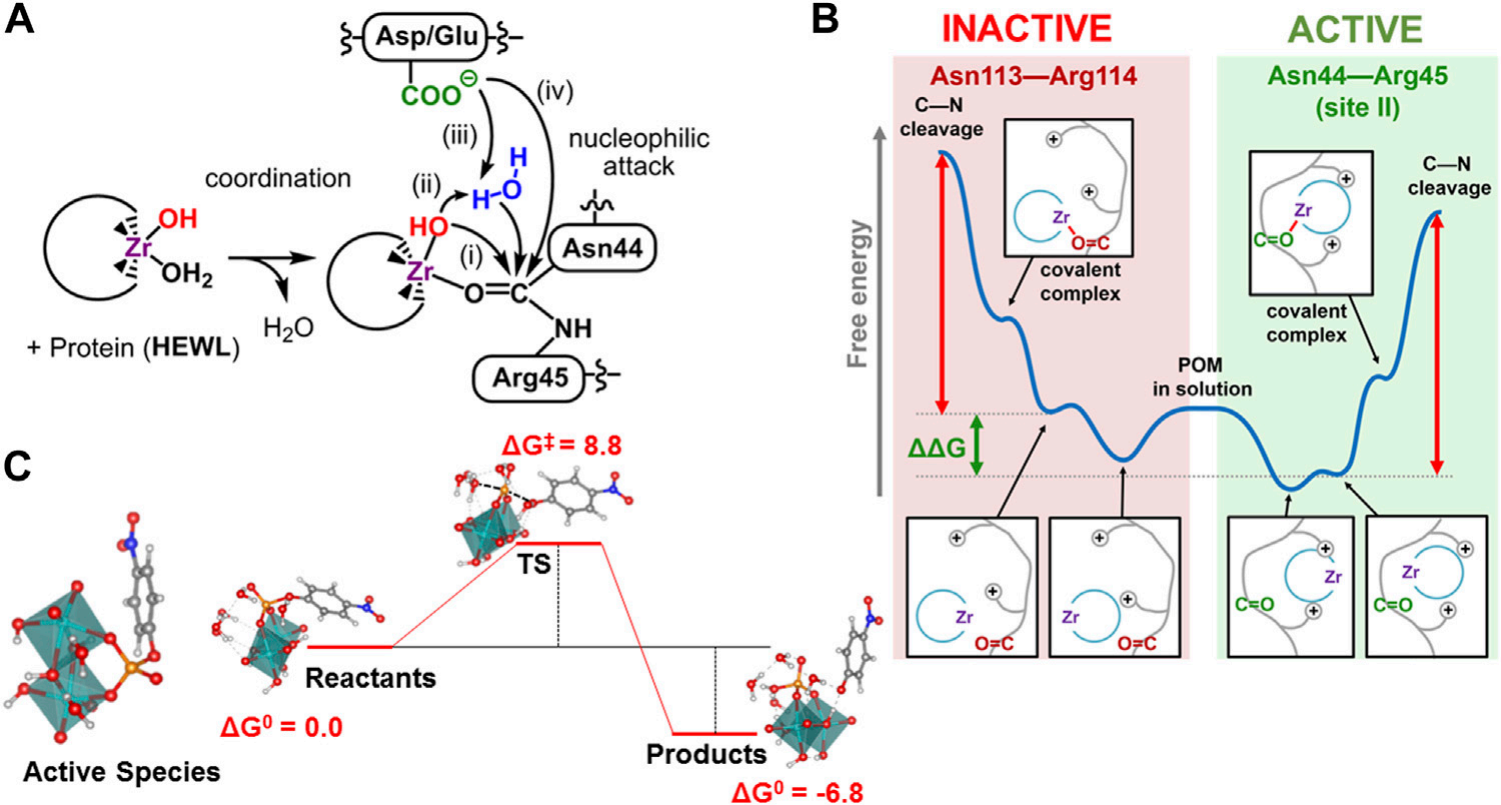
**(A)** Computationally analysed mechanisms for the peptide bond hydrolysis by Zr-substituted POMs: *i*) inner-sphere, *ii*) outer-sphere ligand-assisted, *iii*) outer sphere carboxylate-assisted, *iv*) direct carboxylate attack; **(B)** pictorial representation of the origin of selectivity for the peptide hydrolysis in HEWL by Zr-substituted POMs *via enzyme-like* recognition; **(C)** Proposed active species [Mo_2_O_8_H_4_]^0^, for the hydrolysis of the phosphoester bond of the pNPP model substrate including four explicit water molecules, and the corresponding Gibbs Free Energy profile (kcal·mol^−1^).

The group of Parac−Vogt tested a series of TM-substituted POMs as catalysts in the hydrolysis of peptide bonds in small dipeptides, and among them, only Zr^IV^−, Ce^IV^− and Hf^IV^−substituted POMs showed hydrolytic activity. (Absillis and Parac-Vogt, 2012). Since then, these type of POMs have been successfully applied to the hydrolysis of dipeptides and oligopeptides, as well as, to the selective hydrolysis of a wide range of proteins (for a recent review see ref (van Rompuy and Parac-Vogt, 2019)). Interestingly, different patterns of hydrolysed sites were observed when moving from one protein to another. Computational studies have focus on the characterization of the reaction mechanism and the rationalization of the observed selectivity. (Ly et al., 2015; Jayasinghe-Arachchige et al., 2019; Ly et al., 2019; Solé-Daura et al., 2020b). In all cases, the reaction initiates by coordinating the hydrolytically active metal ion to the amide oxygen atom of the peptide bond. This coordination polarizes carbonyl group, owing the Lewis acid nature of these metal ions and renders the carbon atom more susceptible to suffer a nucleophilic attack. Then, the mechanism can be classified depending on which oxygen fragment is responsible of the nucleophilic attack (Figure 2A): 1) a hydroxo ligand of the TM *via* inner-sphere attack, 2) an external water molecule *via* outer-sphere attack assisted by the TM-OH moiety, or 3) by the carboxylate group of a neighbour amino acid (mechanism iii), and 4) a carboxylate group nearby *via* direct attack. Finally, the protonation of the amide nitrogen induces the C-N bond cleavage.

Pierloot and coworkers characterised the mechanism for the hydrolysis of several dipeptides catalysed by a tetrazirconium−containing sandwich POM by means of DFT calculations. (Ly et al., 2015). The authors found that the most likely mechanism involves the nucleophilic attack of a solvent water molecule to the carbon of the peptide bond assisted by the carboxylate group of the C−terminus end acting as a Brönsted base (mechanism type *iii*). Note that when the dipeptide is embedded in the protein main chain, the carboxyl terminus group is not necessarily available to assist the outer-sphere mechanism. Prabhakar and coworkers analysed the hydrolysis mechanism for different peptide bonds in human serum albumin (HSA) protein catalysed by [Zr(OH) (PW_11_O_39_)]^4−^ anion using static quantum mechanics/molecular mechanics (QM/MM) calculations in the full molecular system. (Jayasinghe-Arachchige et al., 2019). The authors found that the inner-sphere nucleophilic attack of the Zr-OH ligand (mechanism *i*) is somewhat preferred with respect to the outer-sphere attack of an external water molecule assisted by the Zr-OH moiety (mechanism *ii*) for the Cys392-Glu393 bond. Later, the observed hydrolysis at the Asn44-Arg45 site in the HEWL protein by [W_5_O_18_Zr(H_2_O) (OH)]^3−^ Lindqvist anion was analysed in detail by means of a combination of DFT calculations on cluster models obtained from molecular dynamics (MD) simulations, QM/MM calculations, and metadynamics simulations. (Solé-Daura et al., 2020b). Calculations on solvated full-protein model suggests that both *mechanism i* and *ii* are viable under experimental conditions, since the large radius of the Zr atom allows the strained four-membered ring transition state of mechanism *i* to be close in energy to the six-membered ring transition state of mechanism *ii*. Constrained MD simulations on the coordinated POM-Zr···HEWL complex at Asn44-Arg45 site can discard the carboxylate-assisted mechanisms *iii* and *iv* because the closest COO^−^ moieties are far away from the hydrolysed peptide bond. Conversely, for hemoglobin (Hb), calculations support mechanism *iii* at peptide bonds adjacent to an aspartate residue (Asp—X). (Ly et al., 2019). Interestingly, in protein hydrolysis, the C-N scission step governs the overall catalytic activity, (Jayasinghe-Arachchige et al., 2019; Ly et al., 2019; Solé-Daura et al., 2020b), while in dipeptides, the initial nucleophilic attack was found to be the rate−determining step. (Ly et al., 2015). Probably, this is a consequence of the constrains imposed by the main chain of the protein to the peptide bond.

Early attempts to rationalise the observed selectivity pointed out that the cleavage bonds were located at the vicinity of positively charged patches on the protein surface, and that co-crystals confirmed that POM structures bind preferentially to these sites. (Vandebroek et al., 2018). Computational studies have helped to understand the correlation between the POM···protein interactions and the selectivity exhibited by Zr-substituted POMs, as well as, to identify other important factors. (Ly et al., 2019; Solé-Daura et al., 2020b). For Hb protein, the key factor to explain the selectivity towards Asp-X sites is the mechanism, in which direct intramolecular attack of the Asp COO^−^ group on the amide carbon atom is optimal for Asp-X as compared to related X—Asp, X—Glu and Glu—X bonds. (Ly et al., 2019). To explain that only the Asp-X bonds located at random coil fragments of the protein are hydrolysed, the authors suggested that less rigid parts of the protein backbone should be more amenable to electrophilic activation by the Zr ion. For HEWL protein, constrained MD simulations on POM coordination to reactive and non-reactive sites indicate that the origin of the selectivity can be ascribed to an enzyme-like recognition process. (Solé-Daura et al., 2020b). At the reactive site, the positively charged and polar amino acids traps the POM more strongly reducing the energy expense for reorientation and coordination, shifting down the energy profile, and accelerating the hydrolysis rate (Figure 2B). For HSA protein, the comparison of chemically equivalent sites (Arg-Leu) shows strong non-bonding POM···protein interactions in all of them, while the secondary structure of the protein chain favours the hydrolysis at less rigid, coil regions, where lower overall free-energy barriers were calculated using DFT calculations in cluster models (Solé-Daura et al., 2020b).

### Mechanism of Phosphoester Bond Hydrolysis

The phosphoester bond hydrolysis catalysed by molybdate anions as artificial phosphoesterases has been also studied computationally. (Martins et al., 2021; Lanuza et al., 2021; Sа´nchez-Gonzа´lez et al., 2021). In combination with electrospray ionization mass spectroscopy (ESI-MS) experiments, the obtained results provided a different picture of the mechanism compared to previous experimental studies based on NMR, RAMAN and UV-Vis spectroscopy, obtained for [Mo_7_O_24_]^6−^ species. (Lokeren et al., 2008). It was found that a Mo-oxo binuclear species, generated *in situ*, is the promoter of the important catalytic effect when using the pNPP model molecule as substrate (see Figure 2C). Another interesting finding was that starting either from any simple, mononuclear Mo oxide or from polyoxmetalate [Mo_7_O_24_]^6−^ anion, the system can converge to the same binuclear species formed *in situ* [Mo_2_O_8_H_4_]^0^, which promotes reduction of the energy barrier for the phosphoester bond hydrolysis. This barrier is presumably enhanced by the Coulombic repulsion between the negative phosphate environment of the substrate and pair of electrons or the eventual negative charge of the nucleophile, a water molecule or OH^−^. In binuclear [Mo_2_O_8_H_4_]^0^ species the charge is null what would facilitate the approximation of the catalyst to the negative charged phosphate fragment, reducing the energy expense to reach the transition state. This catalytic process shows a low energy barrier as compared to any of the proposed classical mechanisms, substate-assisted and solvent-assisted, for the non-catalysed mechanism. (Duarte et al., 2015; Duarte et al., 2016). At this point, it must be mentioned that some hydrogen bond of the bridge OH of the [Mo_2_O_8_H_4_]^0^ species with the pNPP substrate seems to be involved in the stabilization of the transition state.

The mechanism characterised for the binuclear [Mo_2_O_8_H_4_]^0^ species generated *in situ* from the [Mo_7_O_24_]^6−^ was also investigated for the [W_7_O_24_]^6−^ species acting as catalyst. For the tungstate, it was observed that not only the activation barriers for the hydrolytic process are higher than those for the Mo counterparts but also the obtained products are less stable from a thermodynamic point of view. This behaviour for the hydrolysis of the phosphoester bond in the presence of the W-oxo species comes in hand with the previous experimental results (Cartuyvels, 2008) in which the authors did not observe any catalytic activity in the presence of the [W_7_O_24_]^6−^ analogue.

## Conclusion

This comprehensive review shows that since the early docking studies locating the binding sites of POMs on the surface of proteins, there has been a considerable progress in the computational analysis of the interactions between POMs and biological systems. Incorporating a range of computational tools such as MD simulations, QM/MM and QM/MD methods including metadynamics simulations, or DFT calculations on cluster models, researchers have made possible to provide atomistic description of the binding of POMs to biomolecules, and mechanistic insight into the hydrolysis of peptide and phosphoester bonds with POMs acting as metalloenzymes. Thus, the nature of non-bonding POM···protein interactions has been characterised, showing that the protein affinity depends on charge, size, shape of the POM, as a result of a delicate balance with POM···solvent interactions. Simulations have identified the preferred binding sites for several proteins, reveling in some cases that the specific interaction can be a function of POM composition. By sacrificing molecular details, coarse-grained MD simulations were able to analyse the interaction of POMs with more complex biological systems such as lipid bilayers.

Understanding the factors which govern the activity and selectivity of processes with POMs acting as metalloenzymes is more challenging. However, plausible reaction mechanisms have been proposed for the hydrolysis of peptide and phosphoester bonds catalysed by Zr-substituted POMs and molybdates, respectively. Moreover, in the former case, three factors influencing the selectivity were reported: the specific nature of the dipeptide bond, the secondary structure of peptide chain, and the strong electrostatic-type POM···protein interactions. Although the number of examples is still limited, we are confident that in the coming years the computational studies on the biological activity of POMs will grow significantly becoming an important subarea of computational bioinorganic chemistry. We expect that it will grow the interest for exploring the interaction with biomolecules not only of classical early-transition metal based POMs (Mo, W, or V) but also of the so-called *noble POMs* including less toxic metals such as Au or Pt. Computational studies will expand to analyse the interplay with other biomolecules such as carbohydrates, steroids, triglicerides, etc. Another important topic for biomedicine is the selective affinity of POMs towards one or another biomolecule that would have consequences on their use as eventual drugs targeting specific molecules.
